# Automatic Processing of Nasal Pressure Recordings to Derive Continuous Side-Selective Nasal Airflow and Conductance

**DOI:** 10.3389/fphys.2018.01814

**Published:** 2019-01-07

**Authors:** Lorenz M. Urner, Malcolm Kohler, Konrad E. Bloch

**Affiliations:** ^1^Department of Respiratory Medicine, University Hospital of Zurich, Zurich, Switzerland; ^2^Zurich Center of Integrative Human Physiology, University of Zurich, Zurich, Switzerland

**Keywords:** nasal airflow, nasal resistance, rhinomanometry, rhinitis, noninvasive monitoring, nasal prong pressure transducer, physiological monitoring, signal processing

## Abstract

Monitoring of nasal airflow and conductance provides crucial insights into the variable nature of the nasal resistance, nasal cycle, and ventilation. We have previously shown that tracking of pressure swings at the entrance of each nasal passage by a dedicated catheter system allows bilateral monitoring of nasal airflow over several hours but requires complex linearization and calibration procedures. Side-selective nasal conductance is derived from linearized and calibrated bilateral nasal pressure swings and corresponding driving pressure, i.e., the transnasal pressure difference derived from an epipharyngeal catheter. Manual analysis of such recordings and computation of instantaneous conductance as the ratio of flow to driving pressure over several hours is extremely tedious, time consuming, and therefore not suitable for routine practice. To address this point, we developed and validated a software for automatic processing of nasal and epipharyngeal pressure recordings as a convenient tool for studying the nasal ventilation. The software applies an eight-parameter logistic model to transform nasal pressure swings into side-selective estimates of airflow that are calibrated and further processed along with epipharyngeal pressure to compute bilateral nasal conductance over consecutive, user-selectable time-segments. Essential processing steps include (1) offset correction, (2) low-pass filtering, (3) cross-correlation, (4) cutting of signals into individual breaths, (5) normalization, (6) ensemble averaging to obtain a mean pressure signal for each nasal side, (7) derivation of airflow, conductance, and further variables. Among four evaluated algorithms for calculation of nasal conductance, the derivative of the airflow-pressure curve according to the mean value theorem agreed closest with the gold standard, i.e., the conductance derived from airflow measured by a pneumotachograph attached to an oral-nasal mask and transnasal pressure. In combination with the nasal catheter system, our novel software represents a valuable tool for use in clinical practice and research to conveniently investigate nasal ventilation and its changes occurring spontaneously or in response to various exposures and therapeutic interventions.

## Introduction

Impaired nasal breathing caused by nasal obstruction compromises the quality of life during daytime and sleep (Craig et al., 1998). The manifold causes of nasal obstruction, such as rhinitis or anatomic abnormalities require accurate diagnostic tools to track the highly variable changes of nasal ventilation (Flemons et al., 1999; Kohler et al., 2007, 2009).

Accurate measurements of nasal ventilation are difficult to perform in an unobtrusive way. In clinical practice rhinomanometry is widely used to assess nasal resistance over short periods of time, i.e., over a few breaths. Unfortunately, this standard technique is not suitable for monitoring of nasal ventilation over longer time periods or during sleep, because it requires hand-held instrumentation and special maneuvers to assess patency of the nasal passage (Cole et al., 1989; Hirschberg, 2002). In healthy individuals, the nasal ventilation is changing periodically from a left to a right side predominance, a phenomenon termed the nasal cycle (Kahana-Zweig et al., 2016; Hsu and Suh, 2018). In patients with nasal obstruction due to anatomical alterations or inflammatory diseases, among others, the nasal cycle may be absent, or reduced or the total nasal resistance may be increased resulting in discomfort and a feeling of dyspnea. An ideal diagnostic method for evaluation of nasal ventilation would therefore allow to study the awake or asleep patient over prolonged time periods to capture the variability and side predominance of nasal ventilation. Such a technique would consist of two components: (i) an elaborate measurement instrumentation being unobtrusive, bilateral, patient unresponsive, of minimal instrumentation, applicable during sleep, and suitable for continuous recording, and (ii) an automated signal processing and analysis program capable of analyzing nasal breathing data recorded over several hours (e.g., overnight) with high accuracy and temporal resolution delivering characteristic descriptors of nasal ventilation such as side-selective and total nasal conductance (*Gn*) and airflow. Because processing of large recordings from overnight measurements (a recording time of 6 h at a frequency of 50 Hz results in 10^6^ data points) inevitably precludes manual editing and evaluation, automated computer algorithms have to be used for data processing and analysis.

We have previously described an unobtrusive technique for continuous side-selective monitoring of nasal pressure (Thurnheer et al., 2001; Thurnheer and Bloch, 2004). The technique consists of recording the nasal pressure by a specially designed catheter system at the entrance of each nasal passage, which can be used—after suitable transformation—as a measure of airflow through this passage. In studies of a flow model and human subjects we have shown that pressure at the entrance of the nose can serve as a measure of flow if the relation between pressure swings at the nose and airflow is linearized and a calibration factor determined. This linearization of pressure and airflow was achieved by means of a lookup table, which omits the need of fitting mathematical functions (e.g., square-root transformation), and accurate values for nasal airflow can be derived from nasal pressure signals recorded during sleep (Kohler et al., 2005, 2006). Through the simultaneous recording of the epipharyngeal pressure, side-selective nasal conductance can be calculated (according to *Gn* = flow/pressure). Our system has been successfully applied for the investigation of the effect of impaired nasal ventilation on sleep quality and sleep related breathing disturbances, or for the study of the effects of pharmacological therapies on breathing (Clarenbach et al., 2008).

However, several limitations applied to the evaluation of the recorded nasal pressure data and prevented its use in clinical practice: (i) the manual processing of large datasets using standard spreadsheet software was tedious and time consuming allowing evaluation of a small fraction of the collected data only (e.g., three consecutive breaths every hour were analyzed only); (ii) the transformation of side-selective nasal pressure into airflow using a lookup table could not handle transformation of pressure data lying outside the range of the lookup table; (iii) the conductance calculation was not easily feasible, because additional calculations based on the derived-flow and pressure data were necessary.

As the accurate, complete, and highly resolved tracking of the variable bilateral nasal conductance would provide new insights and support the advancement of sleep respiratory diagnostics and therapeutics, a computer-assisted automatic method to continuously analyze and evaluate nasal pressure recordings is desirable. Therefore, we set out to develop a software program capable of reading and processing side-selective nasal and epipharyngeal pressure recordings. During an initial calibration phase of a few breaths, the program also reads simultaneous airflow recordings measured by a flow meter attached to an oral-nasal mask. Based on these data, the program constructs lookup tables or performs a curve fitting to obtain a linearization of the pressure/airflow relationship. This allows to continuously convert side-selective nasal pressure recordings from multi-hour measurements into airflow, and calculate side-selective nasal conductance taking the epipharyngeal pressure as the driving pressure. Herein, we describe the signal processing principles and validation of the software using nasal pressure recordings of five volunteers. The results of these studies may serve as a basis for a further evaluation of the technique and its application in various clinical and research settings.

## Methods

### Measurement and Recording Devices

#### Side-Selective Nasal Pressure Acquisition by an Unobtrusive Monitoring Method

**Figure 1:**
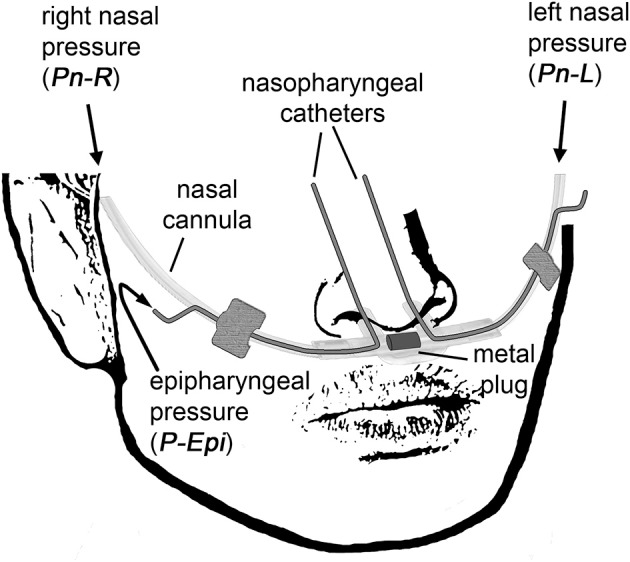
The customized catheter system for non-invasive continuous side-selective monitoring of nasal airflow and conductance. The measured signals are left nasal pressure (*Pn*_L_), right nasal pressure (*Pn*_R_), and epipharyngeal pressure (*P*_Epi_).

Side-selective nasal pressure was recorded using an unobtrusive technique as described previously (Thurnheer et al., 2001; Kohler et al., 2006). It involves the use of a modified nasal prong, which contains a plug blocking the connection midway between the left and right cannulas, thereby allowing to independently record left (*Pn*_L_) and right nasal pressure (*Pn*_R_) by means of differential pressure transducers connected to the left and right nasal cannulas, respectively (Figure 1). Two small-bore catheters were introduced co-axially, one through each nasal cannula, and advanced into the epipharynx during application to measure the epipharyngeal pressure (*P*_*Epi*_) by one common differential pressure transducer.

These signals were then used for the derivation of the side-selective nasal conductances (*Gn*_L_ and *Gn*_R_) and total conductance (*Gn*_L+R_), respectively. Nasal conductance is defined as nasal airflow $\overset{\cdot }{V}$ (obtained from pressure-derived left and right nasal airflow *Fn*_L_ and *Fn*_R_, respectively) divided by the transnasal pressure Δ*P* (obtained from measuring the epipharyngeal pressure referenced to atmosphere).

$$\begin{array}{l} Gn=\frac{\overset{\cdot }{V}}{\Delta P}=Gn_{L}+Gn_{R}=\frac{Fn_{L}+Fn_{R}}{P_{Epi}} \end{array}$$

#### Calibration Procedure

Before and after each nasal pressure and flow measurement, the relationship between side-selective nasal airflow and pressure was established by a calibration procedure. During calibration, a mask with a pneumotachograph attached (Spiroson, ultrasound transit time flowmeter, NDD, Zurich, Switzerland; Buess et al., 1986), was placed over the nose, on top of the nasal cannula. During the occlusion of one naris, airflow and pressure were recorded over a few breaths. The procedure was then repeated for the other naris. Processing and merging of both calibration curves (obtained pre- and post-measurement) by our software program gave a model for the linearization of the pressure-airflow relationship, which was then used to convert the bilateral nasal pressure into airflow during prolonged monitoring periods.

### Computer-Assisted Automatic Signal Processing

#### Software Development for Automatic Processing of Nasal Pressure and Flow Recordings

The software for automatic processing of side-selective nasal pressure recordings was developed using the graphical programming language G within the LabVIEW programming environment from National Instruments, LabVIEW 2014, version 14.0.1 (National Instruments, Austin, Texas, USA). The software was developed and tested on a portable personal computer (PC) running Microsoft Windows® 7 (version 6.1, Service Pack 1), equipped with an Intel® Core™ i7 2.67 GHz processor, 4 GB RAM, and >340 GB free hard disk space. Prior evaluation of algorithms for airflow and conductance calculation from bilateral pressure recordings was carried out with MATLAB 9.1, The MathWorks, Inc., Natick, MA, USA. Essential modules of the software are depicted in Supplementary Figures 1–4 in order to allow reproduction and implementation based on the commercially available LabVIEW software.

### Validation Studies

#### Volunteers

For the present study, we considered the nasal pressure and airflow data of five healthy volunteers (30–41 years old, 1 woman) monitored during sleep. The monitoring experiments had been conducted and published as part of previous studies (Kohler et al., 2006). The protocol was approved by the Cantonal Ethics Committee Zurich. All subjects gave written informed consent in accordance with the Declaration of Helsinki. For the purpose of validating our signal processing program, we used the original, unprocessed data files obtained from our instrumental setup, and reanalyzed the recorded raw data by the signal processing program described below.

#### Data Collection

*Pn*_L_, *Pn*_R_, and *P*_Epi_ were measured continuously during overnight sleep studies (5–7 h of recording time). Although the nasal mask is not required during side-selective nasal flow and epipharyngeal pressure monitoring by the cannula system described above, the mask with the attached pneumotachograph was left in place during the validation studies to obtain total nasal airflow (*F*_PNT_) as a reference standard. Calibration of the pressure-airflow relationship was performed twice, at the beginning and end of the overnight measurements. All respiratory signals were digitally sampled at 50 Hz and stored as comma-separated value (CSV) files amenable to computer-assisted signal processing.

#### Automatic Linearization of Bilateral Nasal Pressure-Airflow Signals

The calibration data, which consists of corresponding pressure and airflow signals of about four consecutive breaths recorded for each nasal side separately while the other nasal side was occluded, was loaded and processed by the calibration module of our nasal signal processing program to generate a calibration file. This file was subsequently readout by the second module of the signal processing program for the automatic translation of side-selective nasal pressure recordings into airflow.

The pairs of corresponding pressure/airflow-calibration data were automatically processed by application of the following signal processing steps (see also the next section for additional details): (1) offset correction, (2) low-pass filtering, (3) cross-correlation between corresponding pairs of left and right pressure/airflow signals, (4) cutting of the signals into individual breaths, (5) normalization to obtain ensembles of breaths of same length, (6) ensemble averaging to obtain a mean pressure/airflow signal for each nasal side, (7) fitting of a calibration curve to the pressure/airflow data for each nasal side, (8) storing the resulting curve-fit as a CSV-file.

To identify the pressure-airflow transformation that gave the most accurate results, we implemented three different algorithms in the calibration module. These algorithms are either based on the fitting of a mathematical function or on the computation of a smoothed curve to each left and right nasal airflow-pressure signal:

1. Fitting a smoothed curve by a lowess algorithm (Cleveland and Devlin, 1988). The resulting smoothed curve was stored as a lookup table containing discrete pressure/airflow-value pairs.
2. Fitting a polynome of user-defined order using the least squares method.
3. Fitting the following 8-parameter logistic (8-PL) model (Bewick et al., 2005) in the least squares sense by a Levenberg-Marquardt (Levenberg, 1944; Marquardt, 1963) algorithm:
  $$\begin{array}{lll} Fn_{L,R} & = & \;\frac{\left(a+b*Pn_{L,R}\right)-\left(c+d*Pn_{L,R}\right)}{e+\text{exp}\left(-f*\left(Pn_{L,R}-g\right)\right)}\\ + & \left(c+d*Pn_{L,R}\right)+h \end{array}$$

where *Fn*_L,R_ is left or right nasal airflow derived from corresponding nasal pressure *Pn*_L,R_; a, b, c, and d determine the upper and lower asymptote of the logistic curve; e, f, g, and h determine different curve characteristics, such as the offset along the *Fn*-axis (h) or *Pn*-axis (g, point of maximum growth), or the sigmoidal shape (e, and f, growth rate).

#### Automatic Derivation of Bilateral Nasal Airflow and Conductance From Nasal Pressure Recordings

*Pn*_L,R_ recorded during sleep of 5–7 h were automatically loaded and processed by the second module of our signal processing program to generate side-selective *Fn*_L,R_ and conductance *Gn*_L,R_. The automatic translation of pressure into airflow was achieved by the calibration curve generated by the first module (vide supra). The bilateral conductance was computed from the derived airflow according to a user-specified algorithm as explained in more detail below.

Due to the long duration of recordings, which would make graphical representation unpractical, mean airflow, and conductance were calculated over user-specified consecutive time segments (bins). *Pn*_L_,_R_ and *P*_Epi_, and for validation purposes *F*_PNT_, were automatically processed by the following steps performed for each time bin: (1) offset correction, (2) low-pass filtering, (3) cross correlation of (optional) *F*_PNT_ along *P*_Epi_, (4) cutting into individual breaths by the following algorithm: (i) detection of start and end points of each breath for each pressure signal (*Pn*_L_, *Pn*_R_, and *P*_Epi_) by the LabVIEW Basic Level Trigger Detection VI, (ii) removal of any odd triggers, (iii) merging of all triggers of all pressure signals into one single array of triggers, (iv) all pressure signals were cut into individual breaths along the trigger positions, (5) normalization, (6) ensemble averaging to obtain mean pressure curves for consecutive time segments (bins), (7) derivation of left and right nasal airflow *Fn*_L,R_ from the pressure signals by means of the user-specified calibration method, (8) calculation of the left and right nasal conductance *Gn*_L,R_ by a user-specified algorithm, (9) computation of additional breathing parameters [e.g., tidal volume (*VT*), respiratory rate, ratio of left to right nasal airflow], (10) saving of all time-binned pressure signals, derived airflow, and conductance signals to a CSV-file.

In order to assess the automatic calculation of pressure-derived conductance, several algorithms were implemented which calculate the bilateral conductance according to the following formulas:

$$\begin{array}{l} 1)Gn_{L,R}=mean\left(\frac{F{n^{\prime }}_{\;L,R}}{P_{Epi}}\right)\\ 2)Gn_{L,R}=mean_{25\%}\left(\frac{F{n^{\prime }}_{\;L,R}}{P_{Epi}}\right)\\ 3)Gn_{L,R}=\;\frac{Fn_{L,R}\left(P_{Epi,max}\right)-Fn_{L,R}\left(P_{Epi,min}\right)}{P_{Epi,max}-P_{Epi,min}}\\ 4)Gn_{L,R}=mean\left(\frac{dF{n^{\prime }}_{\;L,R}}{dP_{Epi}}\right) \end{array}$$

Algorithms 1, 2, and 4 initially fit the 8-PL model (described above) to the plot of *Fn*_L,R_ vs. *P*_Epi_ to obtain a smoothed airflow curve *Fn'*_L,R_; from the resulting fit, either the mean of airflow divided by *P*_Epi_ (Algorithm 1), the trimmed mean (the lowest and highest 12.5% are discarded, Algorithm 2), or the mean of the analytically determined slope of *Fn*_L,R_ for each value of *P*_Epi_ (Algorithm 4) is calculated. Algorithm 3 calculates the slope of the plot of *Fn* against *P*_Epi_ by applying the mean value theorem.

#### Data Analysis

The accuracy of the airflow and conductance, obtained by the different calibration and calculation methods as implemented in our automatic signal processing software, was determined by comparison of the derived total flow *Fn*_L+R_ to *F*_PNT_. All data streams were processed by the same automatic procedures to estimate the derived total conductance *Gn*_L+R_ and reference conductance *Gn*_PNT_. Averaged periods of breaths of *F*_PNT_ with a *VT* of < 200 mL or >800 mL (attributed to mouth breathing, mask leaks, or recording/processing errors) were excluded from the comparison analysis of airflow and conductance.

#### Statistical Analysis

Agreement between two methods was determined by calculation of the bias (mean difference) and 95% limits of agreement (LOA, the range of bias ± 1.96 *SD*) (Bland and Altman, 1986). All statistical evaluations were carried out with MATLAB 9.1, The MathWorks, Inc., Natick, MA, USA.

## Results

### Design and Implementation of the Signal Processing Program

**Figure 2:**
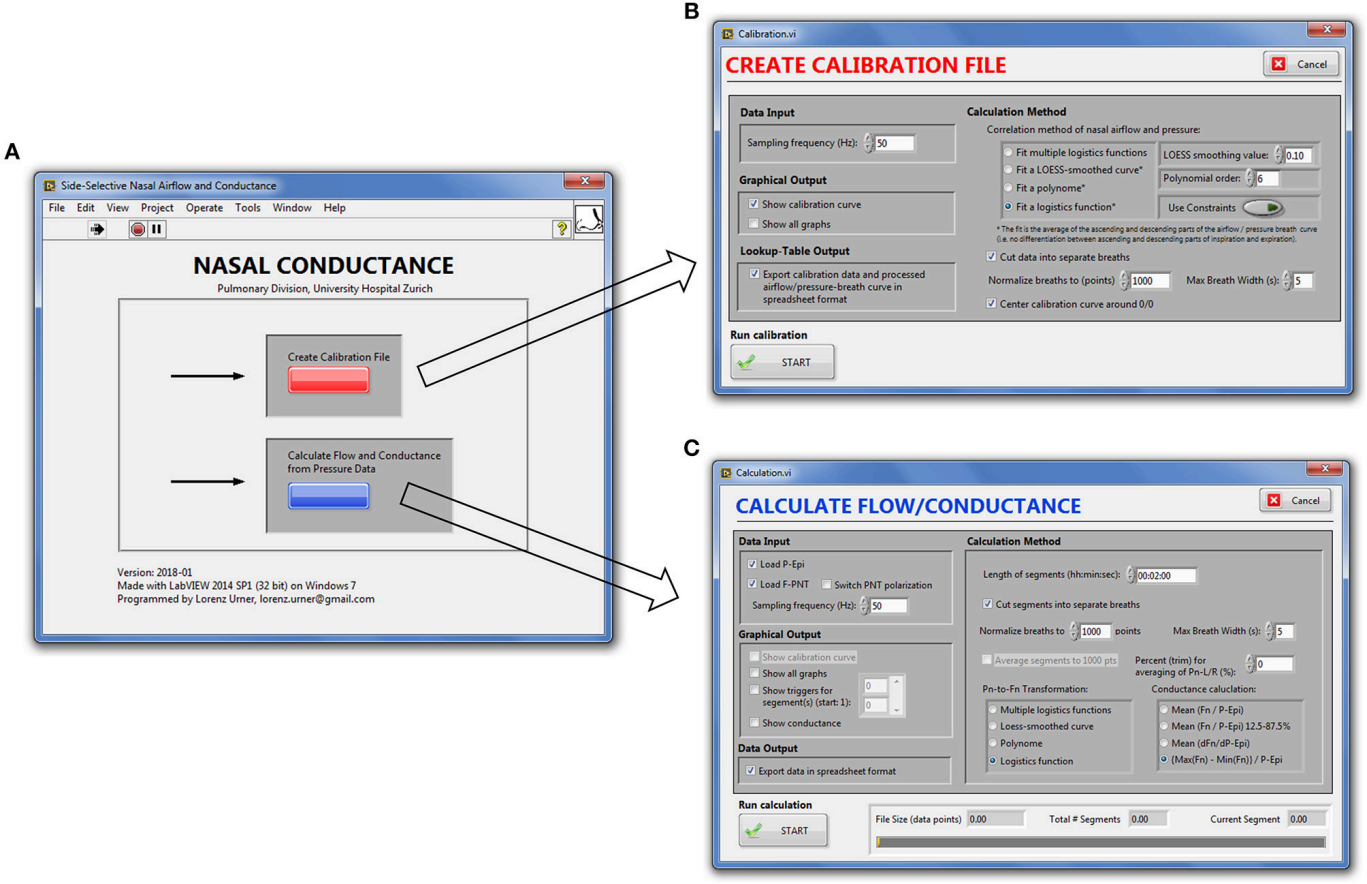
Graphical user interface of the nasal pressure and airflow processing program. **(A)**, Main window. **(B)**, Calibration module. **(C)**, Module for derivation of bilateral airflow and conductance from nasal pressure recordings.

Our signal processing program for automatic, continuous transformation of bilateral pressure into airflow and conductance comprises two modules: the first module calibrates side-selective nasal pressure with airflow; the second module carries out the actual processing of bilateral nasal pressure recordings into airflow and conductance according to the calibration data generated by the first module. These two modules can be accessed from the main graphical user interface, which is displayed after startup of the program (Figure 2).

We implemented a modular program architecture using LabVIEW's virtual instruments (VIs) for each signal processing task (e.g., offset correction, low-pass filtering, conductance calculation) and connecting them in series (LabVIEW block diagrams are depicted in the Supporting Information (SI), Supplementary Figures 1–4). Through case-structures the user can choose between different algorithms for the same task (e.g., generation of the calibration curve) and switch on or off graphical output after each processing step. Depending on the chosen level of detail (i.e., duration of the bins of averaged breaths) and conductance algorithm, signal processing of up to 6 h of continuously recorded data took between 10 and 180 s as evaluated on our computer system.

#### Algorithms for Automatic Calibration of Nasal Pressure/Airflow Transformation

**Figure 3:**
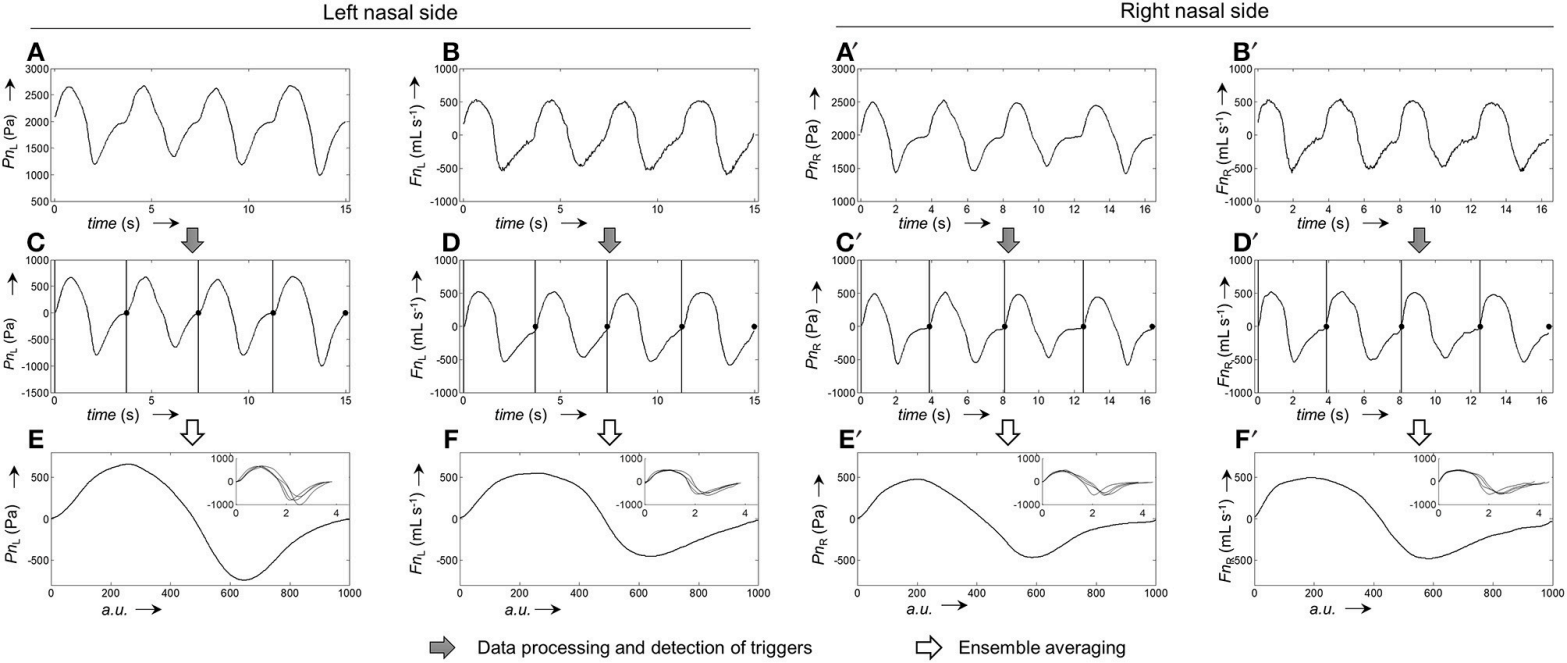
Representative example for the generation of pressure and airflow signals during one averaged breath used for the calibration of left and right nasal sides. **(A,A')**, Raw recordings of left and right nasal pressure, respectively. **(B,B')**, Raw recordings of left/right nasal airflow. **(C,C')**, Left/right nasal pressure recordings after application of signal correction steps (filtering, offset correction, cross-correlation); the vertical bars and black dots, respectively, indicate the positions at which the signals were cut into the individual breaths (vertical lines: start of breath, dots: end of breath). **(D,D')**, Left/right nasal airflow recordings after application of signal correction steps (same as for pressure recordings). **(E,E')**, Ensemble average of the left/right nasal pressure during one breath, normalized to 1,000 data points. **(F,F')**, Ensemble average of left/right nasal airflow during one breath, normalized to 1,000 data points. The ensemble averaged curves were obtained from the individual breaths shown as overlays in the respective insets.

**Figure 4:**
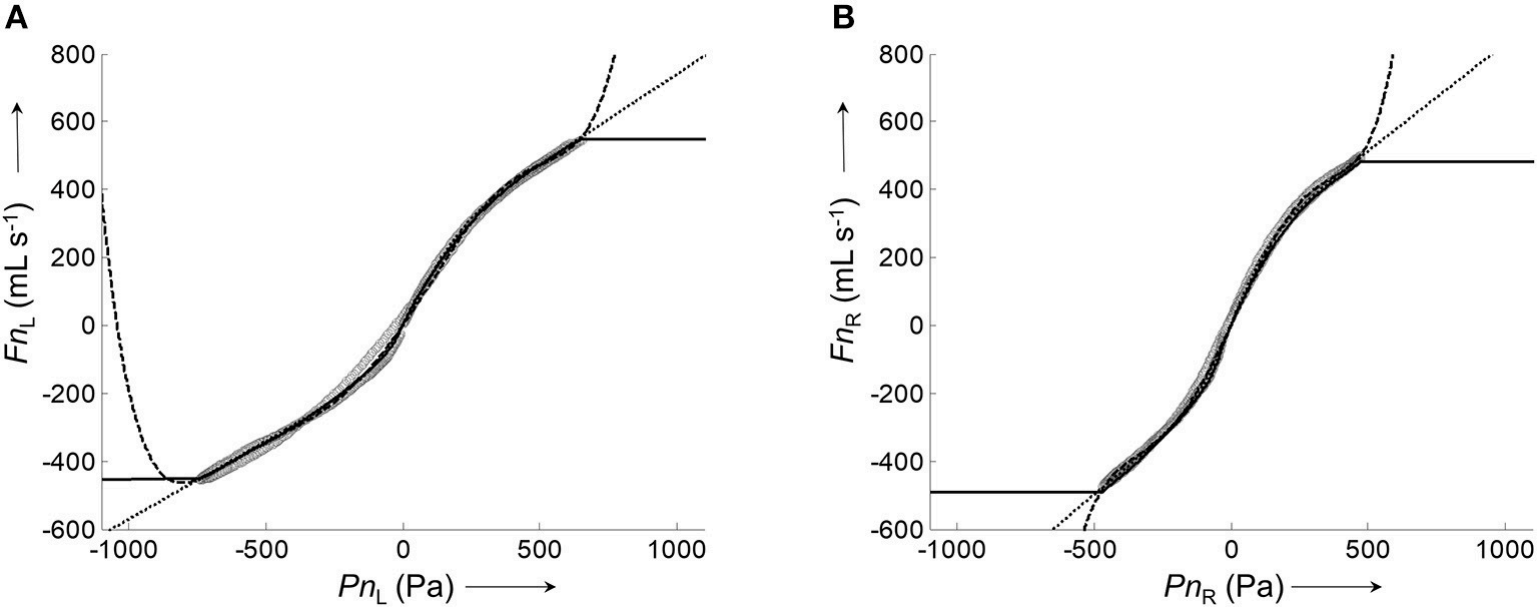
Representative calibration curves for the left and right nasal side fitted by different algorithms to the corresponding pairs of average airflow and pressure signals. **(A)**, Plot of the average left nasal airflow (data shown in Figure 3F) against the average left nasal pressure (data shown in Figure 3E). **(B)**, Plot of average right nasal airflow (data shown in Figure 3F') against the average right nasal pressure (data shown in Figure 3E'). Calibration curves were fitted to the data (gray circles) by different algorithms: lowess smoother (solid), polynomial fit (dashed), and 8-PL function (dotted).

The calibration process for the generation of average pressure/airflow curves during one breath from raw calibration pressure/airflow data is displayed in Figure 3. The resulting calibration curves obtained from the different fitting algorithms are shown in Figure 4.

All assessed algorithms (lowess, polynomial, 8-PL model) gave calibration curves which closely reproduced the measured airflow during the recorded pressure range. However, only the calibration curve based on the 8-PL model extrapolated the course of airflow in a physiologically meaningful way, whereas the other calibration models (lowess and polynomial fit) produced inadequate airflow values in the extrapolated pressure range.

Therefore, the 8-PL model was chosen for the airflow/pressure linearization and generation of calibration files, which were subsequently employed for the automatic derivation of bilateral airflow and conductance from long-term pressure recordings as reported below.

#### Derivation of Continuous Bilateral Nasal Airflow and Conductance From Pressure Recordings

**Figure 5:**
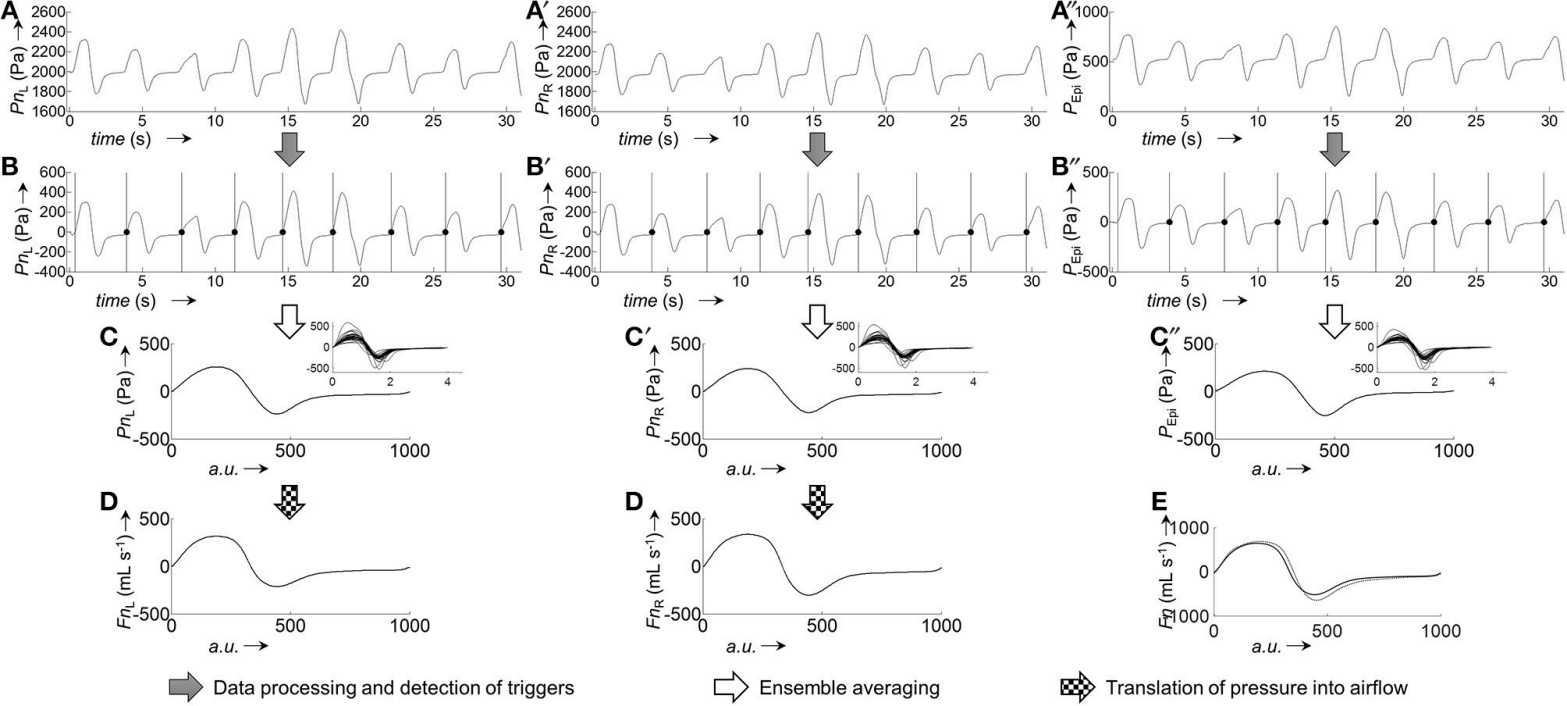
Derivation of ensemble averaged bilateral airflow (*Fn*_L_, *Fn*_R_) and epipharyngeal pressure (*P*_Epi_) from bilateral pressure recordings (*Pn*_L_, *Pn*_R_) of a representative 2-min segment. Excerpt (first 30 s) of raw pressure recordings of a 2-min segment: **(A)**, left nasal pressure (*Pn*_L_); **(A')**, right nasal pressure (*Pn*_R_); **(A”)**, epipharyngeal pressure (*P*_Epi_). Processed pressure signals (after application of filtering, offset correction, and cross-correlation): **(B)**, *Pn*_L_; **(B')**, *Pn*_R_; **(B”)**, *P*_Epi_; the vertical bars and black dots indicate the positions at which the signals were cut into the individual breaths (vertical bars: start of breath, dots: end of breath). Ensemble averaged pressure: **(C)**, *Pn*_L_; **(C')**, *Pn*_R_; **(C”)**, *P*_Epi_. The insets show the individual breaths, overlayed on top of each other, from which the ensemble averaged curves were obtained. Pressure-derived airflow: **(D)**, *Fn*_L_, **(D')**, *Fn*_R_. **(E)**, Overlay of total derived airflow (*Fn*_L+R_, solid line) and airflow from a flow meter (*F*_PNT_, dotted line). All ensemble averaged signals were normalized to 1,000 data points.

**Figure 6:**
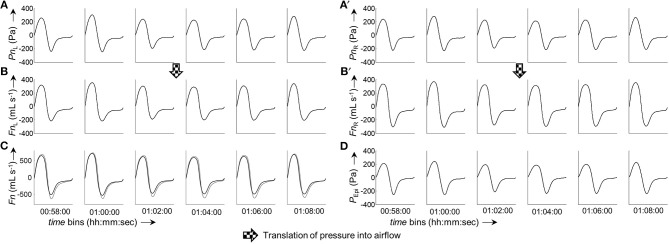
Excerpt from a 6 h measurement. The panels show a consecutive series of ensemble averaged bilateral nasal pressure and derived airflow sorted in 2-min groups. **(A,A')**, Averaged left and right nasal pressure, respectively. **(B**,**B')**, Averaged left and right nasal airflow, respectively. **(C)**, Overlay of total derived airflow (*Fn*_L+R_, solid line) and airflow measured by a flow meter (*F*_PNT_, dotted line). **(D)**, Epipharyngeal pressure (*P*_Epi_). All ensemble averaged curves were normalized to 1,000 data points. The time given under each column of bins refers to the start time of the respective bin.

Continuous bilateral derived nasal airflow (and subsequently conductance) was obtained by grouping the bilateral pressure, which was recorded during sleep (~10^6^ data points per channel), into time segments (bins) of user-defined length (e.g., 2 min). Figure 5 illustrates the processing of raw pressure recordings into ensemble averaged pressure curves per time bin, and derivation of airflow by means of the 8-PL calibration model. The output of our program are a series of averaged pressure and derived airflow grouped into time segments of user-defined length (Figure 6). The close, continuous tracking of airflow—obtained from data collected by our catheter system—in comparison with airflow measured by a flow meter is shown for a representative series of time segments in Figure 6C.

**Figure 7:**
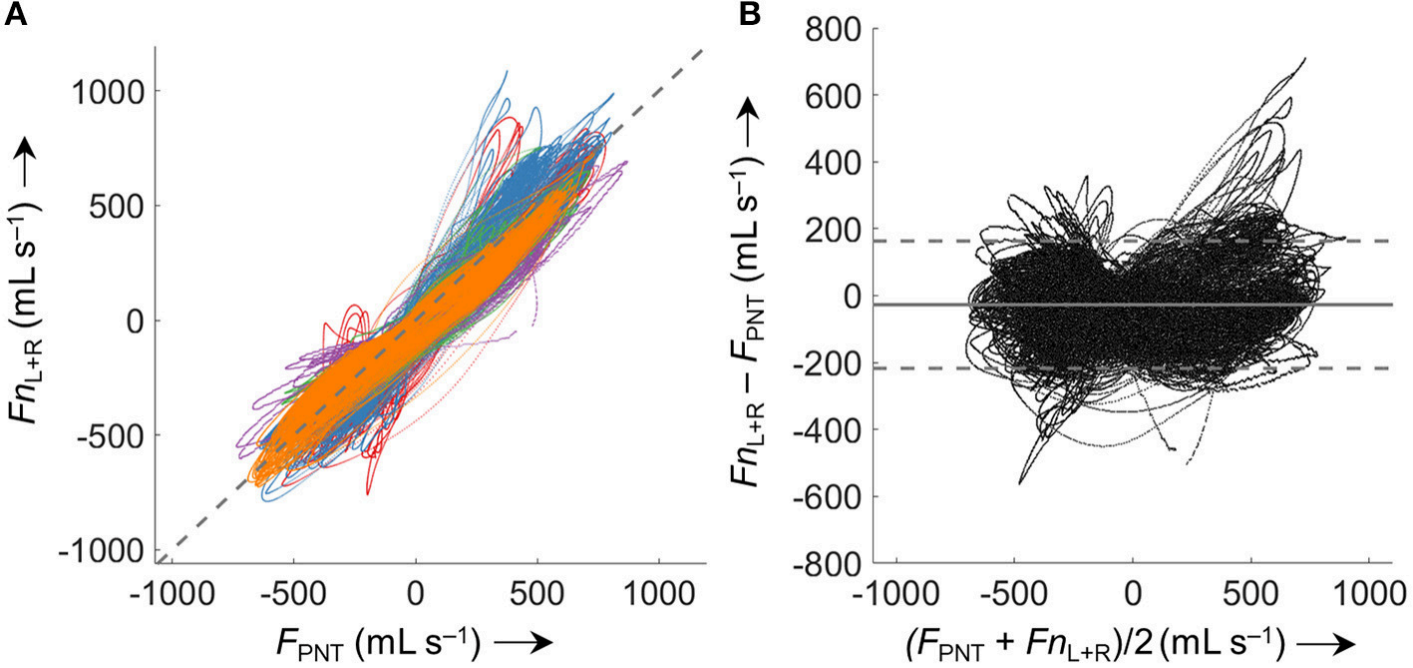
Comparison of derived (*Fn*) and measured airflow (*F*_PNT_). **(A)** Identity plot of left and right nasal pressure derived nasal airflow (*Fn*_L+R_) vs. values measured by a flow meter (*F*_PNT_). The dashed gray line indicates identity. **(B)** Bland-Altman plot; the solid line represents the mean difference (bias), dashed lines represent limits of agreement (±1.96 SD). Values from 5 analyzed subjects (different colors) were included (563'000 paired measurements). Averaged periods of breath with a *VT* <200 mL or >800 mL (attributed to mouth breathing, mask leaks, or recording/processing errors) were excluded from the comparison.

Comparison of *Fn*_L+R_ with *F*_PNT_ for all five analyzed data sets (5.63 ^*^ 10^5^ paired measurements) according to Bland-Altman (Figure 7) resulted in a bias ± LOA of −27 ± 190 mL s^−1^.

Side-selective nasal conductance was obtained from the derived airflow according to four different algorithms. The implemented algorithms calculate either a mean value (mean or trimmed mean) of the conductance determined at every epipharyngeal pressure per time segment according to $Gn=\frac{Fn^{\prime }}{P_{Epi}}$ (*Fn*' is a smoothed airflow curve obtained by 8-PL fitting), or the derivative of the curve *Fn* vs. *P*_Epi_ is determined (as an average or according to the mean value theorem).

**Figure 8:**
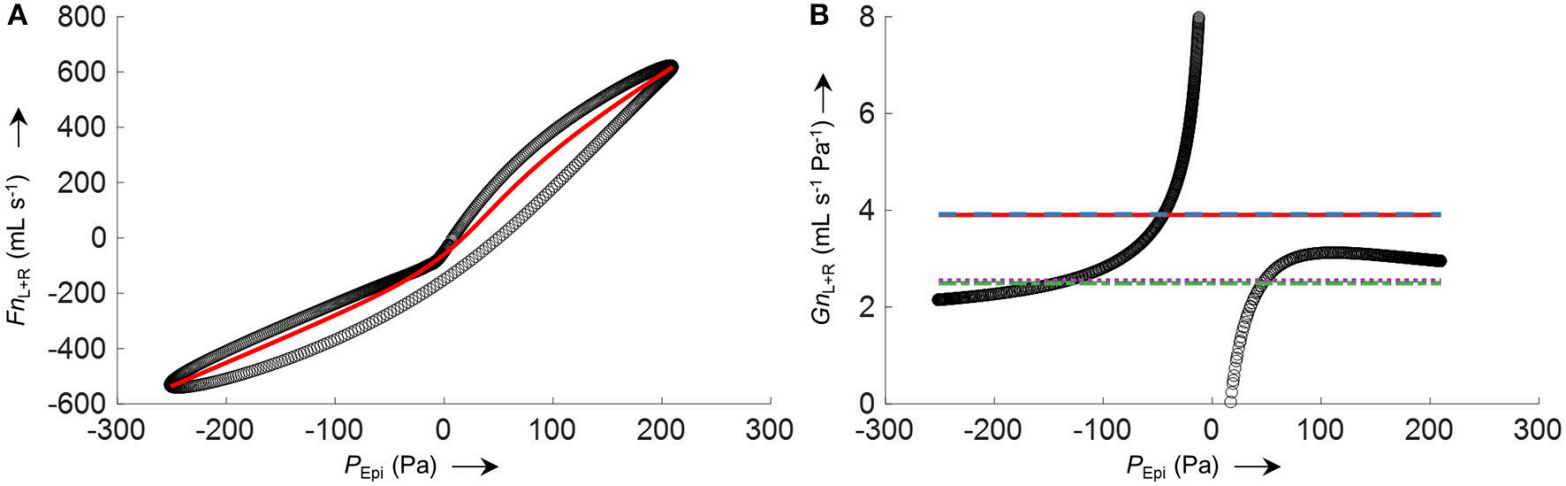
Example for the calculation of total nasal conductance (*Gn*_L+R_) of a representative 2-min segment from averaged nasal airflow (*Fn*_L+R_) and epipharyngeal pressure (*P*_Epi_) by different conductance algorithms. **(A)** Non-linear logistic curve fit (8-PL model, red line) to the averaged left and right nasal airflow *Fn*_L+R_ plotted against *P*_Epi_ (black circles). **(B)** $\frac{Fn_{L+R^{\prime }}}{P_{Epi}}$ (black circles) where *Fn'*_L+R_ corresponds to left and right airflow derived by the non-linear curve fit shown in **(A)**; average total nasal conductance *Gn*_L+R_ obtained by different algorithms are depicted as straight horizontal lines: $mean\left(\frac{Fn_{L+R^{\prime }}}{P_{\text{Epi}}}\right)$ (solid red), $mean_{25\%}\left(\frac{Fn_{L+R^{\prime }}}{P_{Epi}}\right)$ (dashed blue), $\frac{Fn_{L+R}\left(P_{\text{Epi},\text{max}}\right)-Fn_{L+R}\left(P_{\text{Epi},\text{min}}\right)}{P_{\text{Epi},\text{max}}-P_{\text{Epi},\text{min}}}$ (dash-dotted green), $mean\left(\frac{dFn_{L+R^{\prime }}}{dP_{Epi}}\right)$ (dotted purple). The conductance functions show a discontinuity at zero pressure because of a slight hysteresis or dysynchrony of pressure and flow signals.

The process of conductance calculation for a representative 2-min time segment is illustrated in Figure 8A (fitting of an 8-PL curve to the plot of *Fn* vs. *P*_Epi_) and Figure 8B (comparison of conductances obtained by the four different algorithms). As is evident from Figure 8B, conductance algorithms 1/2 and 3/4, respectively, yield almost the same conductance values. The difference is that conductance algorithms 2 and 3 are more robust to outliers or processing errors than their corresponding algorithms 1 and 4, respectively.

**Figure 9:**
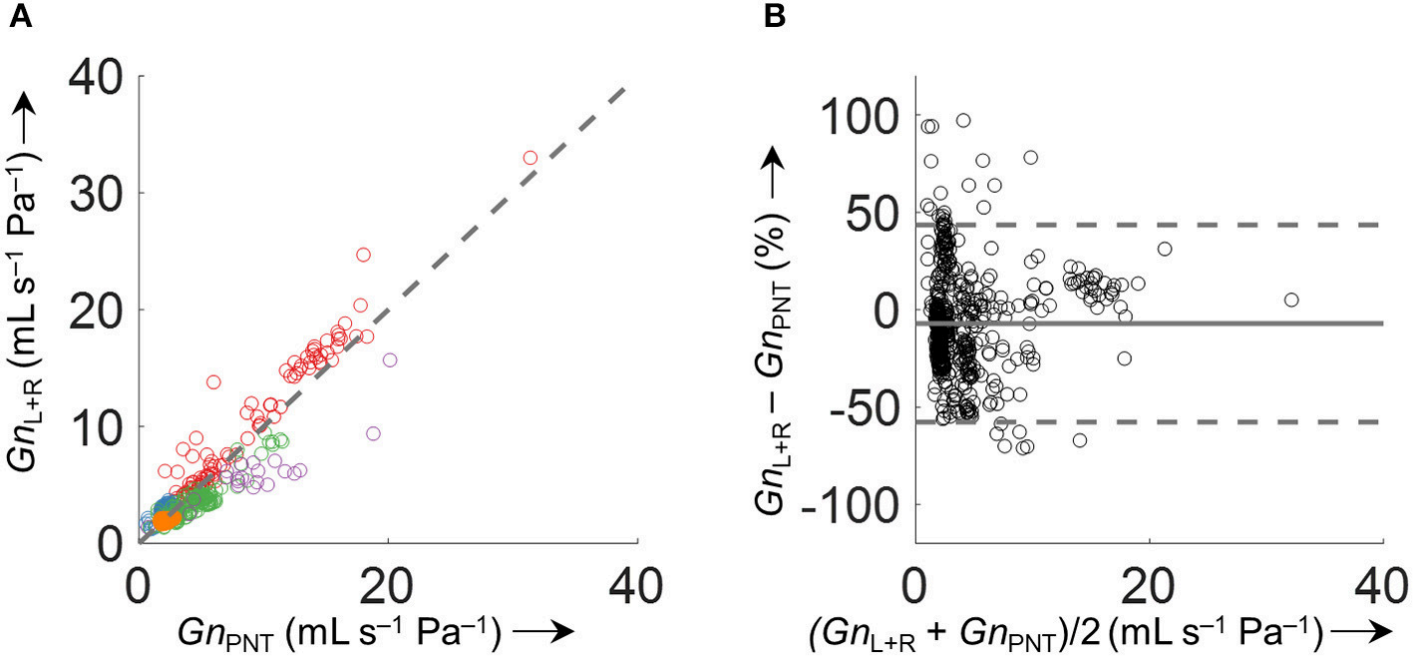
Comparison of derived and measured conductance obtained by $\frac{Fn_{\text{L}+\text{R}}\left(P_{\text{Epi},\text{max}}\right)-Fn_{\text{L}+\text{R}}\left(P_{\text{Epi},\text{min}}\right)}{P_{\text{Epi},\text{max}}-P_{\text{Epi},\text{min}}}$. **(A)** Identity plot of derived total nasal conductance (*Gn*_L+R_) and *Gn*_PNT_. The different colors refer to the five analyzed datasets. The dashed gray line indicates unity. **(B)** Bland-Altman plot. The conductance was calculated as an average over 2 min time intervals (563 data points). Conductance values of time intervals with *VT*_Fn_ or *VT*_PNT_ < 200 mL/s or >800 mL/s and outliers (*Gn*_L+R_−*Gn*_PNT_ > 500%) were excluded from the comparison.

Accuracy of the various conductance algorithms was assessed by comparing the pressured-derived conductance to the conductance derived from a flowmeter. Analysis of all conductance values obtained for all five data sets (560 paired measurements) revealed that algorithm 3 ($\frac{Fn_{L+R}\left(P_{\text{Epi},\text{max}}\right)-Fn_{L+R}\left(P_{\text{Epi},\text{min}}\right)}{P_{\text{Epi},\text{max}}-P_{\text{Epi},\text{min}}}$) resulted in the closest reproduction of conductance with a bias ± LOA of −0.2 ± 2.8 mL s^−1^ Pa^−1^ (−7.1±51%). Validation data for 2 min time segments are shown in Table 1 and Figure 9, and for other time intervals of various lengths (1 to 30 min) in Supplementary Figure 5 in the SI.

**Table 1:** Accuracy of nasal pressure-derived conductance obtained by various algorithms according to Bland-Altman^a^.

**Conductance algorithm**	**Formula for calculation of nasal conductance**	**Bias (mL s^**−1**^ Pa^**−1**^)**	**Limits of agreement(mL s^**−1**^ Pa^**−1**^)**	**Bias(%)**	**Limits of agreement(%)**
1	$mean\left(\frac{Fn^{\prime }{\;}_{\text{L}+\text{R}}}{P_{\text{Epi}}}\right)$	0.3	43	−5.1	133
2	$mean_{25\%}\left(\frac{Fn^{\prime }{\;}_{\text{L}+\text{R}}}{P_{Epi}}\right)$	0.1	5.4	−7.4	65
3	$\frac{Fn_{\text{L}+\text{R}}\left(P_{\text{Epi},\text{max}}\right)-Fn_{\text{L}+\text{R}}\left(P_{\text{Epi},\text{min}}\right)}{P_{\text{Epi},\text{max}}-P_{\text{Epi},\text{min}}}$	−0.2	2.8	−7.1	51
4	$mean\left(\frac{dFn^{\prime }{\;}_{L+R}}{dP_{Epi}}\right)$	−0.2	3.1	−7.7	56

a Conductance values of time segments with a mean VT_Fn_ or VT_FPNT_ < 200 mL or >800 mL and outliers (Gn_L+R_−Gn_PNT_ > 500%) were excluded from the analysis. Limits of agreement = 1.96^*^SD. Values in percent obtained from (Gn_L+R_− Gn_PNT_) / [(Gn_L+R_ + Gn_PNT_)/2].

### Continuous Side-Selective Nasal Conductance From Nocturnal Pressure Recordings

**Figure 10:**
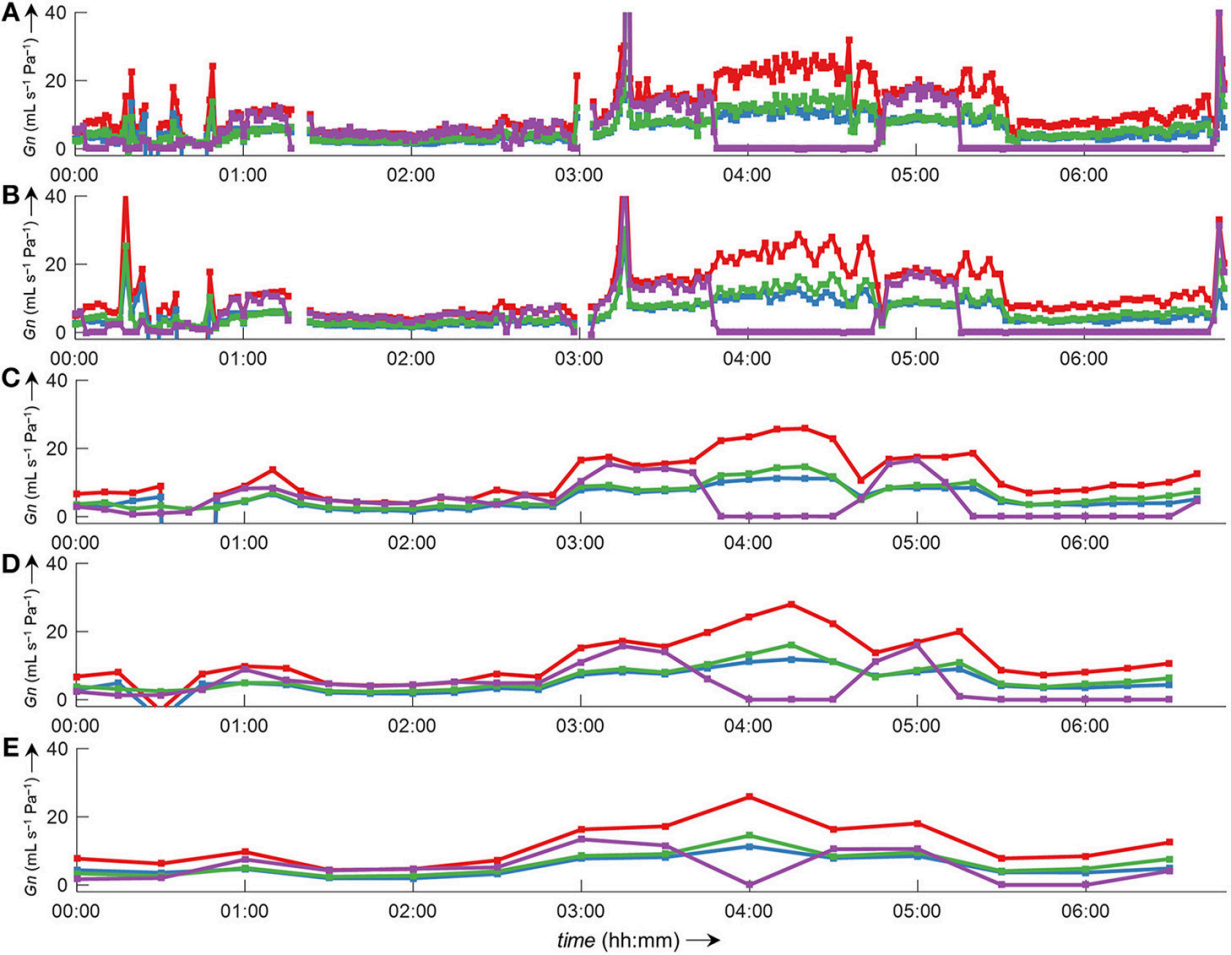
Bilateral pressured-derived conductance curves obtained by conductance algorithm 3 in comparison to conductance obtained from a flow meter during a 7 h overnight measurement evaluated for different consecutive time intervals. Overlay of left conductance (*Gn*_L_, blue line), right conductance (*Gn*_R_, green line), total conductance (*Gn*_L+R_, red line), and conductance derived from a flow meter (*Gn*_PNT_, purple line). The conductance was evaluated as an average value for different time intervals according to formula 3 (Table 1): **(A)**, 1-min intervals; **(B)**, 2-min intervals; **(C)**, 10-min intervals; **(D)**, 15-min intervals; **(E)**, 30-min intervals. Values of zero conductance of *Gn*_PNT_ are attributed to mouth breathing or mask leaks. Outliers and gaps in the curves (missing conductance values) result from processing errors.

**Figure 11:**
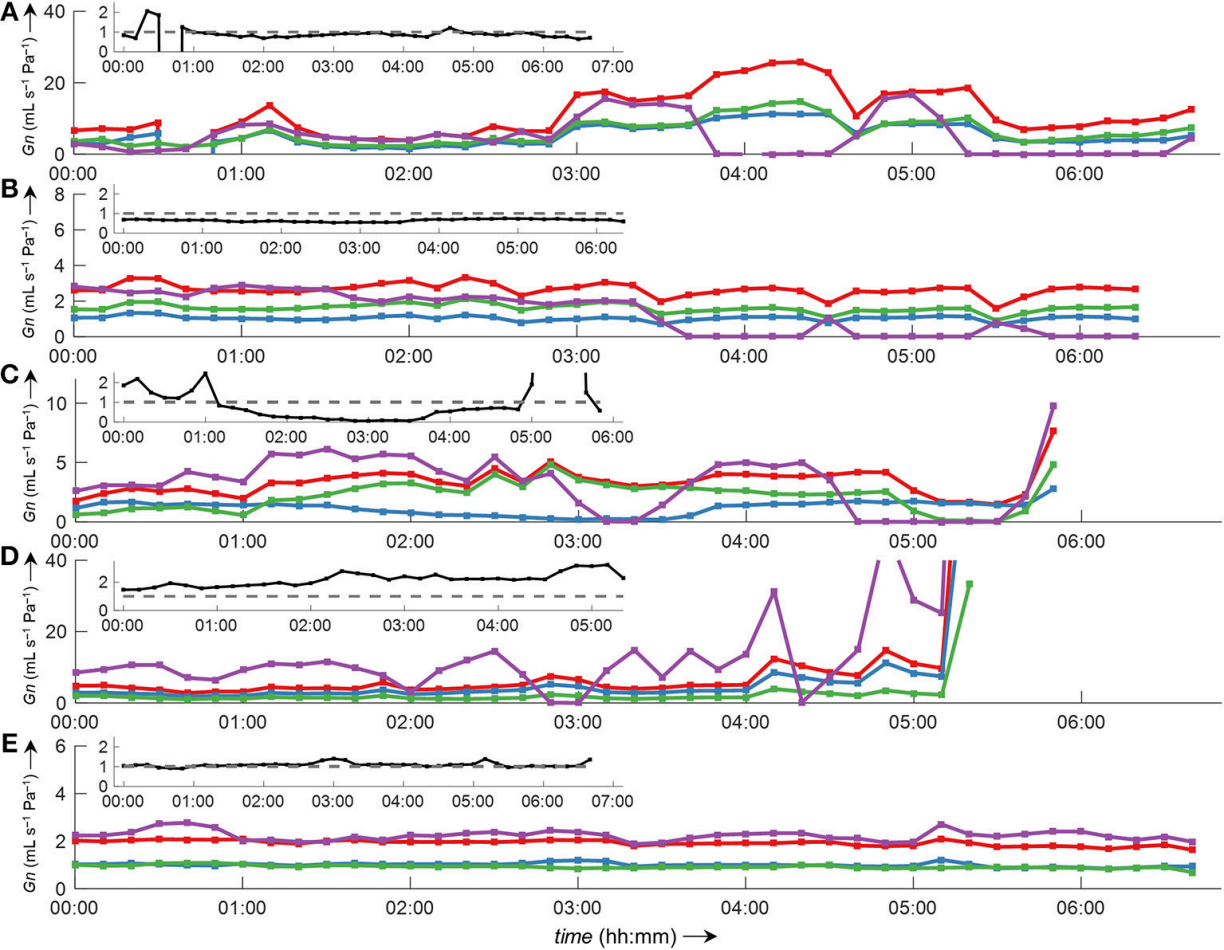
Bilateral conductance curves obtained by algorithm 3 in comparison to conductance obtained from a flow meter during overnight measurements of all five datasets analyzed in this study **(A–E)**. The data were evaluated as an average of 10-min intervals. Overlay of left conductance (*Gn*_L_, blue line), right conductance (*Gn*_R_, green line), total conductance (*Gn*_L+R_, red line), and conductance derived from a flow meter (*Gn*_PNT_, purple line). Outliers and gaps in the curves (missing conductance values) result from displacement of catheters and/or the face mask with attached flow meter during body movements. The insets show the ratio *Gn*_L_/*Gn*_R_ (black line).

Side-selective nasal conductance derived from nasal pressure recordings was calculated for all five analyzed data sets according to the evaluated calibration and conductance algorithms: calibration was performed by fitting the 8-PL function, and conductance was calculated with conductance algorithm 3. The duration of time segments was generally set to 2 min, but other durations (e.g., from one individual breath to 30 min or even longer periods) were also possible and calculations were completed within reasonable computation times. Figure 10 depicts the course of side-selective nasal pressure-derived conductance together with data measured by a flow meter in a representative individual. In this example, conductance was determined as an average for every 1, 2, 10, 15, and 30 min of recording time of this overnight measurement. Figure 11 and Supplementary Figure 5 demonstrate that agreement among nasal cannula-derived and flowmeter-derived values of nasal conductance is similar for various averaging periods (1, 10, 15, 30 min).

## Discussion

Our specially designed catheter system for the unobtrusive recording of side-selective nasal pressure allowed to develop an automatic data analysis tool, capable of computing nasal airflow and conductance in a convenient, robust, and continuous manner. We envisaged to obtain detailed data on the course of side-selective conductance during multi-hour measurements, and as accurate as possible with regard to the employed calibration procedure. However, such a software implementation demanded advanced signal processing and mathematical modeling as described in detail in the Methods section. The final achieved goal was to create a computer program, which can be utilized by clinicians interested in nasal physiology and pathology without the need to understand the detailed signal processing steps.

In this work, we describe the development of a computer program for the automatic processing and transformation of continuous multi-hour bilateral nasal pressure recordings into derived airflow and conductance. We describe a proposed signal processing approach featuring a special 8-PL calibration algorithm for the linearization of nasal pressure and airflow, and we also introduce a novel, simple method for the calculation of nasal conductance based on the derivative of the airflow/pressure relationship. We demonstrate the practical utility of our software program by presenting several examples of nasal airflow and conductance data recorded over the course of multi-hour overnight sleep studies.

### Software Development

We implemented our signal processing program with the LabVIEW development environment due to its very straightforward to use graphical programming style, and because it comprises a large library of predefined functions, such as for reading/writing of data files, waveform analysis/processing, and general algorithms necessary for computation. These functions are so-called LabVIEW Virtual Instruments, and they can be connected together through wires (representing the data streams) in a so-called block diagram by means of the LabVIEW graphical programming language. Especially, graphical user interfaces or display of graphs could thus be produced in a simple manner. However, the implementation of more complicated algorithms, such as the detection and triggering of individual breaths, which contain many nested case structures and loops (selected examples of the block diagrams are depicted in the SI, Figures 1–4), turned out to be cumbersome, and the evaluation of the different conductance algorithms had to be completely carried out in MATLAB. The final program can read and process pressure data of over 6 h in a short time (<3 min). It works without any abnormal program termination, despite highly variable signal shapes, which make recognition of individual breaths difficult. This can lead to false identification/cutting of breaths, and eventually produce outliers during conductance calculation.

### Evaluation of Algorithms for Pressure-Airflow Linearization and Conductance Calculation

Because we measured nasal pressure as a surrogate for airflow, a linearization procedure had to be applied, due to the non-linear relationship between pressure changes detected at the nostrils and actual airflow. A common method to do this is the square-root transformation of pressure signals ($\overset{\cdot }{V}=\sqrt{Pn}$) (Farré et al., 2001; Thurnheer et al., 2001). Our approach is based on a calibration procedure, where simultaneously side-selective airflow and pressure is recorded during a few consecutive breaths. From these data, a lookup table is constructed, which provides a direct translation of nasal pressure into airflow. However, due to the limited duration of the calibration period, we applied a smoothing function (8-PL model), which also extrapolates into the regions outside of the calibration range. The quality of this calibration procedure determines how well the measured pressure will be eventually translated into airflow during the actual measurement. Construction of the lookup table from both calibration events performed before and after the overnight measurement provides more robust results than using only one set of the calibration data (see Supplementary Figure 6 and Supplementary Tables 1, 2 in the SI). A prolonged calibration period might improve the accuracy of pressure-to-airflow conversion.

Conductance is defined as the ratio of flow vs. transnasal pressure. As is evident from Figure 8B, this ratio has a discontinuity at zero pressure (with undefined conductance at this point). Several methods have been used to quantify the highly variable nasal conductance by a single value (*Gn*): e.g., median value (Thurnheer and Bloch, 2004), mean value (Kohler et al., 2006), determination at a special designated pressure (e.g., 75 Pa or 150 Pa during inspiration or expiration) (Nathan et al., 2005) or radius (Broms et al., 1979). However, such specific pressures might not always be achieved during breathing, and can lead to incorrect results as discussed e.g., in Vogt et al. (2016). The methods for conductance calculation analyzed in this study take into account all airflow-pressure-value pairs monitored during one breath, and are therefore applicable to all shapes of airflow-pressure curves. Conductance algorithm 3 showed the best agreement and is computationally less intensive, as no curve fitting/smoothing procedure has to be applied to the airflow-pressure curve as is the case for the other algorithms 1, 2, and 4. Because conductance values are obtained from the derived-airflow, the accuracy of derived-conductance depends in the same way on the goodness of the calibration table as does the derived airflow. Clearly, our program enables the convenient processing of various nasal pressure datasets over various averaging periods with sufficient accuracy and in a short amount of time (Figures 10, 11 and Supplementary Figure 5). In addition, it gives useful complementary information, such as the ratio of left-to-right nasal conductance (Figure 11, insets). However, as stated before, the quality of the derived data critically depends on the calibration step, and if this is not carried out sufficiently, the transformation of nasal pressure into airflow, and conductance will lead to inaccurate results.

### Relevance and Implications of the Continuously Derived Bilateral Airflow and Conductance

Until recently, bilateral nasal airflow and conductance have been measured at discrete time points only, involving uncomfortable instrumentation, special breathing maneuvers requiring patient cooperation and tedious analysis of recordings. This has prevented the widespread use of such measurement in clinical practice. By designing the nasal catheter system along with dedicated software described in the current report we provide the opportunity to researchers and clinicians alike to investigate the nasal pathophysiology in detail during natural breathing over many hours including during nocturnal sleep. The essential components of the signal analysis proposed here comprise the transformation of nasal pressure swings into airflow signals based on a calibration procedure that requires simultaneous recordings by the nasal cannula and a pneumotachograph over a few breaths at the beginning and end of the measurement period. Once the calibration is applied, our technique allows to continuously compute nasal airflow, conductance and derived indices using nasal and epipharyngeal pressure signals. Our approach has advantages over previous attempts to study the nasal cycle (Kahana-Zweig et al., 2016) by providing quantitative, side-selective estimates of nasal airflow, and conductance rather than just nasal pressure swings. The powerful software tool that we have validated in the current study can be utilized to obtain various indices reflecting nasal physiology and the breathing pattern as diagnostic indicators of disease and response to therapeutic interventions. The favorable results of our study warrants a further application of the technique in larger groups of healthy individuals and patients with nasal pathologies of various etiologies.

## Conclusion

Processing of side-selective nasal pressure recordings into airflow and conductance is greatly facilitated by our computer program in comparison to manual processing. Computations are performed in a short time, take into account all recorded pressure data, and give continuous derived airflow and conductance values. The 8-PL model accurately produces pressure to airflow transformation within and outside the calibration range. However, the agreement of derived-airflow with airflow measured by a face mask depends heavily on the quality of the calibration process. A prolonged or repeated calibration process might be beneficial for overall agreement. The herein proposed algorithm 3 for conductance calculation showed best agreement of all evaluated methods, and is also the computationally least intensive. It might therefore be a useful alternative to the other established methods for determination of conductance. The implementation of the described software in combination with the nasal catheter system in a monitoring unit represents a valuable tool for application in clinical practice and research for the evaluation of disturbances of nasal ventilation over time in response to various exogenous or endogenous stimuli.

## Author Contributions

KB was involved with conception and design of this work. LU developed the software for automatic signal processing. All authors were involved with data collection, interpretation, and drafting and revising the article. All authors approved the final version of the manuscript.

### Conflict of Interest Statement

The authors declare that the research was conducted in the absence of any commercial or financial relationships that could be construed as a potential conflict of interest.

## Supplementary Material

Additional Figures and Tables (LabVIEW block diagrams, Bland-Altman analyses of derived conductance for different time intervals, and analyses of data based on one calibration event only).
