# Supplementary material for: Automatic Processing of Nasal Pressure Recordings to Derive Continuous Side-Selective Nasal Airflow and Conductance
Source: Front Physiol. 2019 Jan 7;9:1814. doi: 10.3389/fphys.2018.01814 (PMC6330336; doi:10.3389/fphys.2018.01814)
Supplement: Supplementary file 1 [file Data_Sheet_1.pdf]

Supplementary Material

Automatic Processing of Side-Selective Nasal Pressure Recordings to Derive Continuous Side-Selective Nasal Airflow and Conductance

Lorenz M. Urner, Malcolm Kohler and Konrad E. Bloch*

*** Correspondence:** Konrad E. Bloch: konrad.bloch@usz.ch

1 Supplementary Figures and Tables

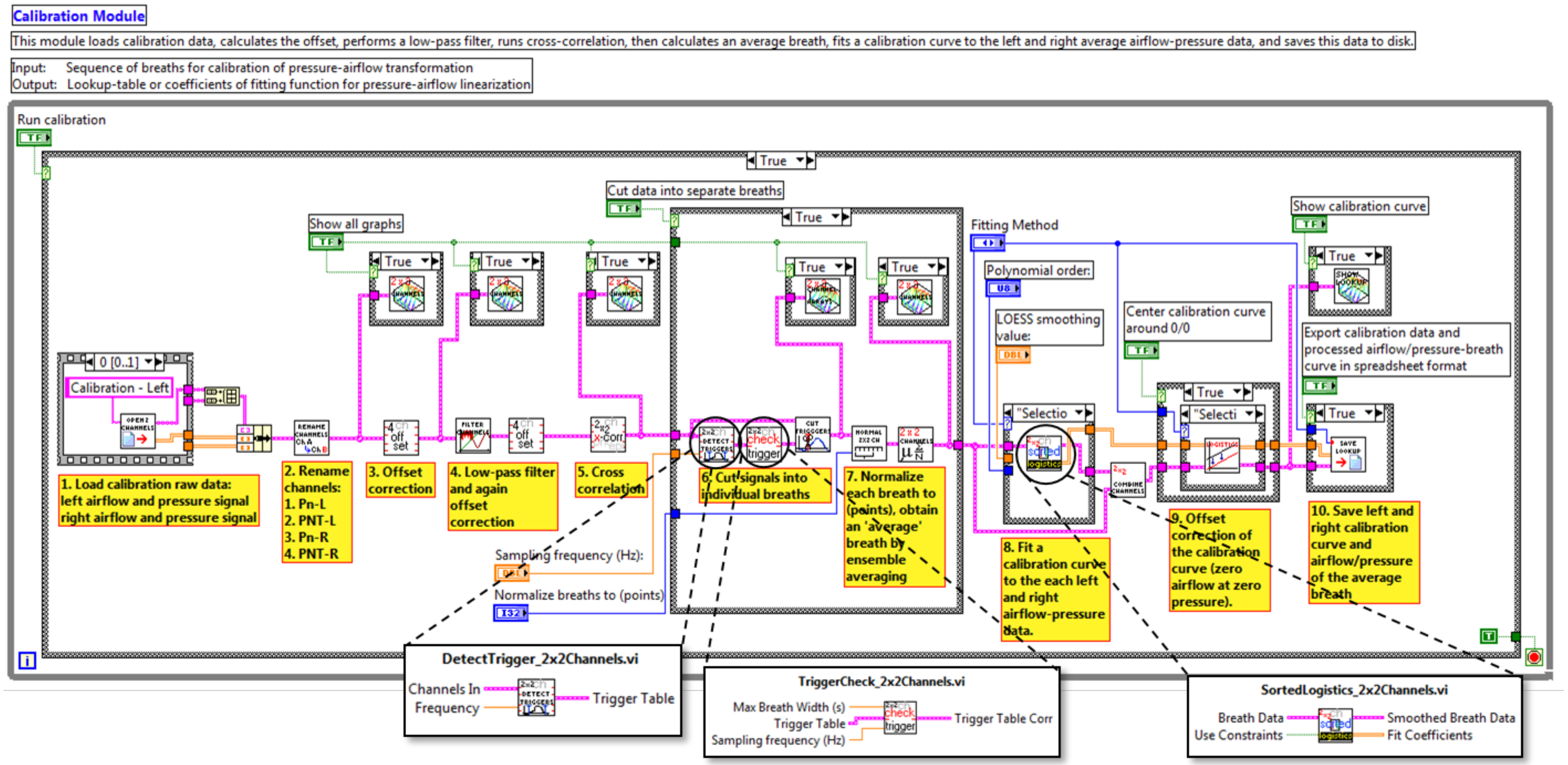

Supplementary Figure 1. LabVIEW block diagram of the first module, which calibrates side-selective nasal pressure with airflow. Three crucial signal processing steps are marked by black circles: detection of individual breaths by the 'DetectTrigger' VI, quality control of the detected triggers (which define the start and end point of a breath) by the 'TriggerCheck' VI, and fitting of a logistic calibration curve by the 'Sorted Logistics' VI. The LabVIEW block diagrams of these VIs are depicted in **Supplementary Figure 2**.

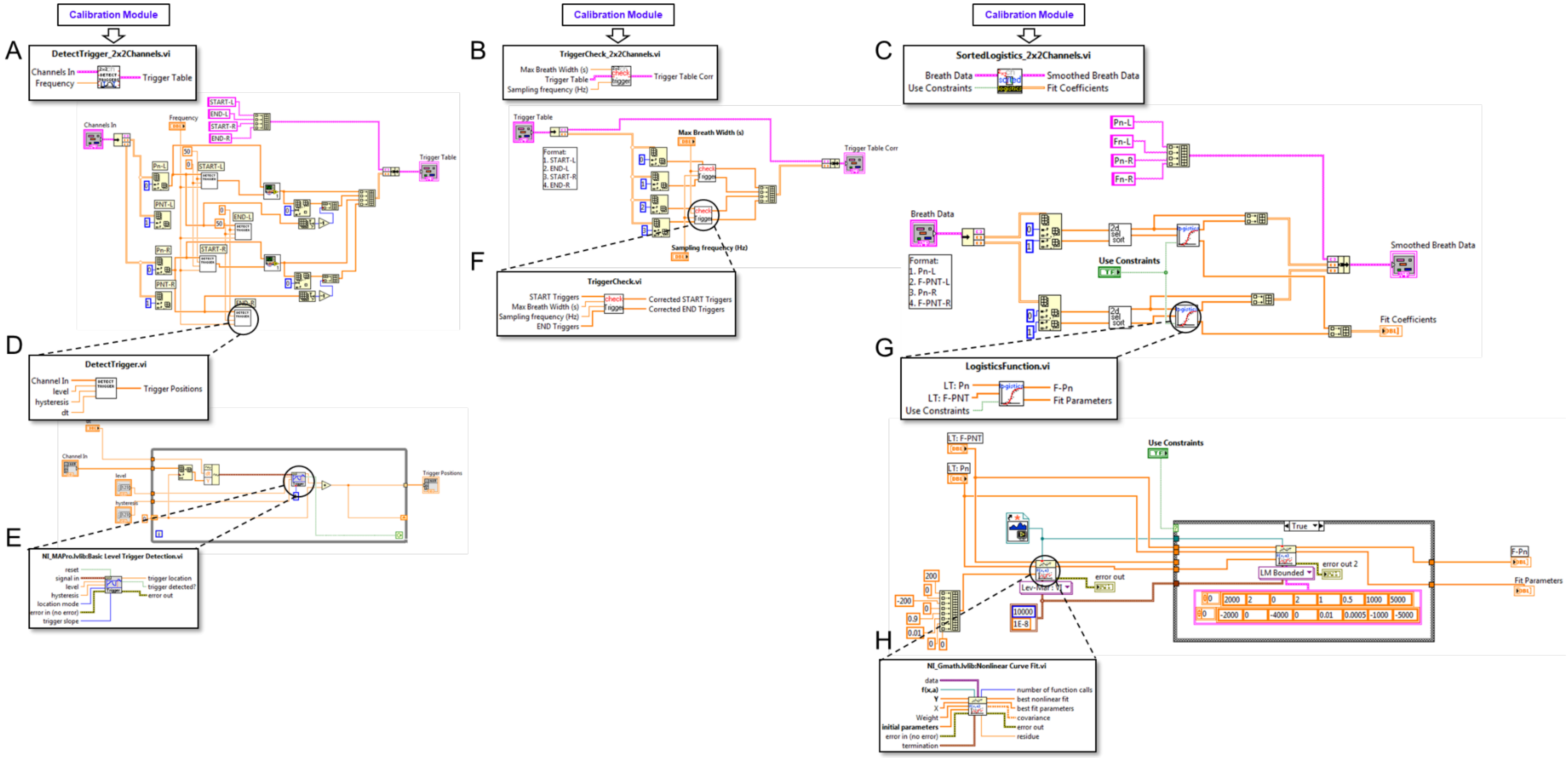

Supplementary Figure 2. LabVIEW block diagrams of three crucial signal processing steps, which are part of the calibration module shown in **Supplementary Figure 1**. **(A)**, 'DetectTrigger' VI. **(B)**, 'TriggerCheck' VI. **(C)**, 'Sorted Logistics' VI. All these VIs contain additional nested sub-VIs, which in turn can again contain nested sub-sub-VIs as depicted in **(D)** to **(H)**.

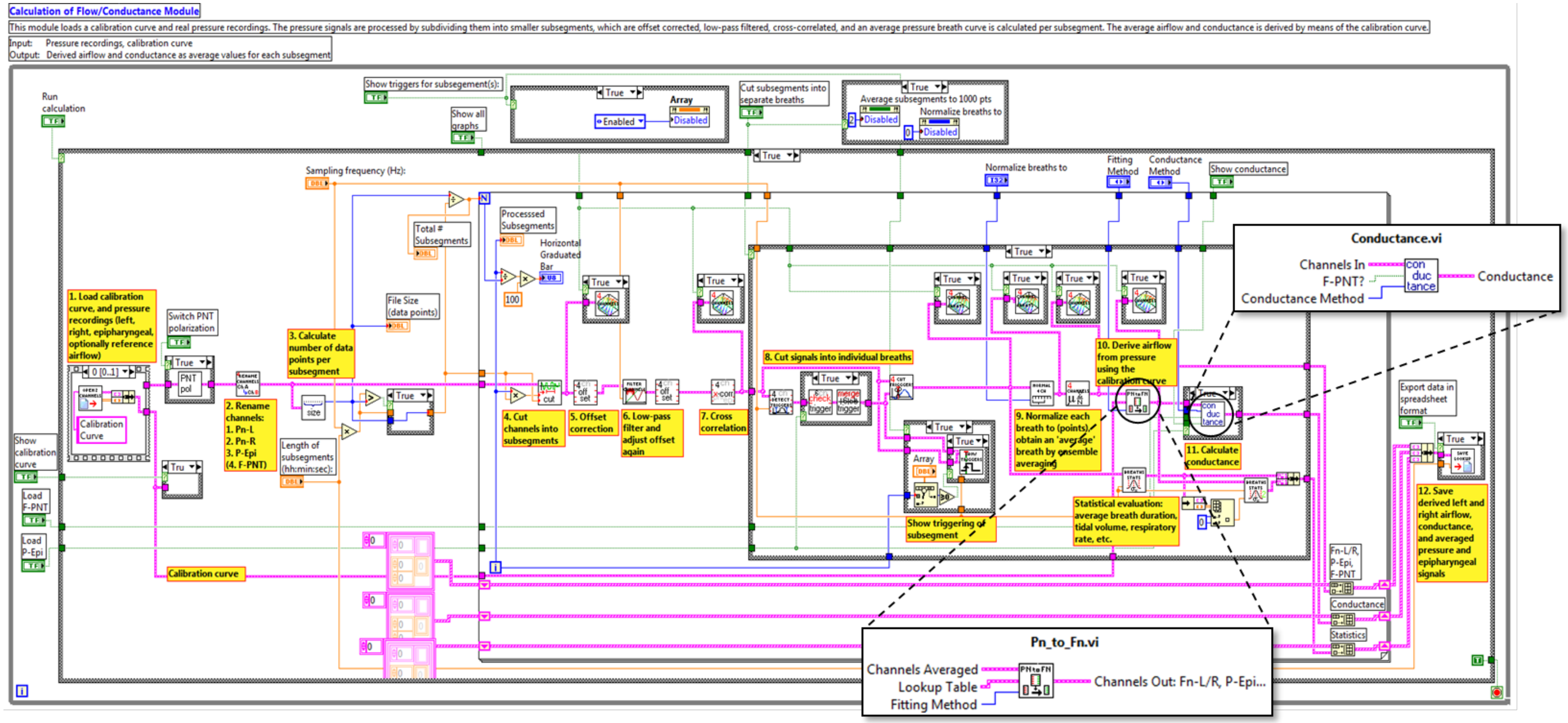

Supplementary Figure 3. LabVIEW block diagram of the second module, which processes bilateral nasal pressure recordings into derived airflow and conductance based on the calibration data generated by the first module. Two crucial signal processing steps are marked by black circles: translation of side-selective nasal pressure into airflow by the ‘Pn_to_Fn’ VI, and calculation of the nasal conductance by the ‘Conductance’ VI. The LabVIEW block diagrams of these VIs are depicted in **Supplementary Figure 4**.

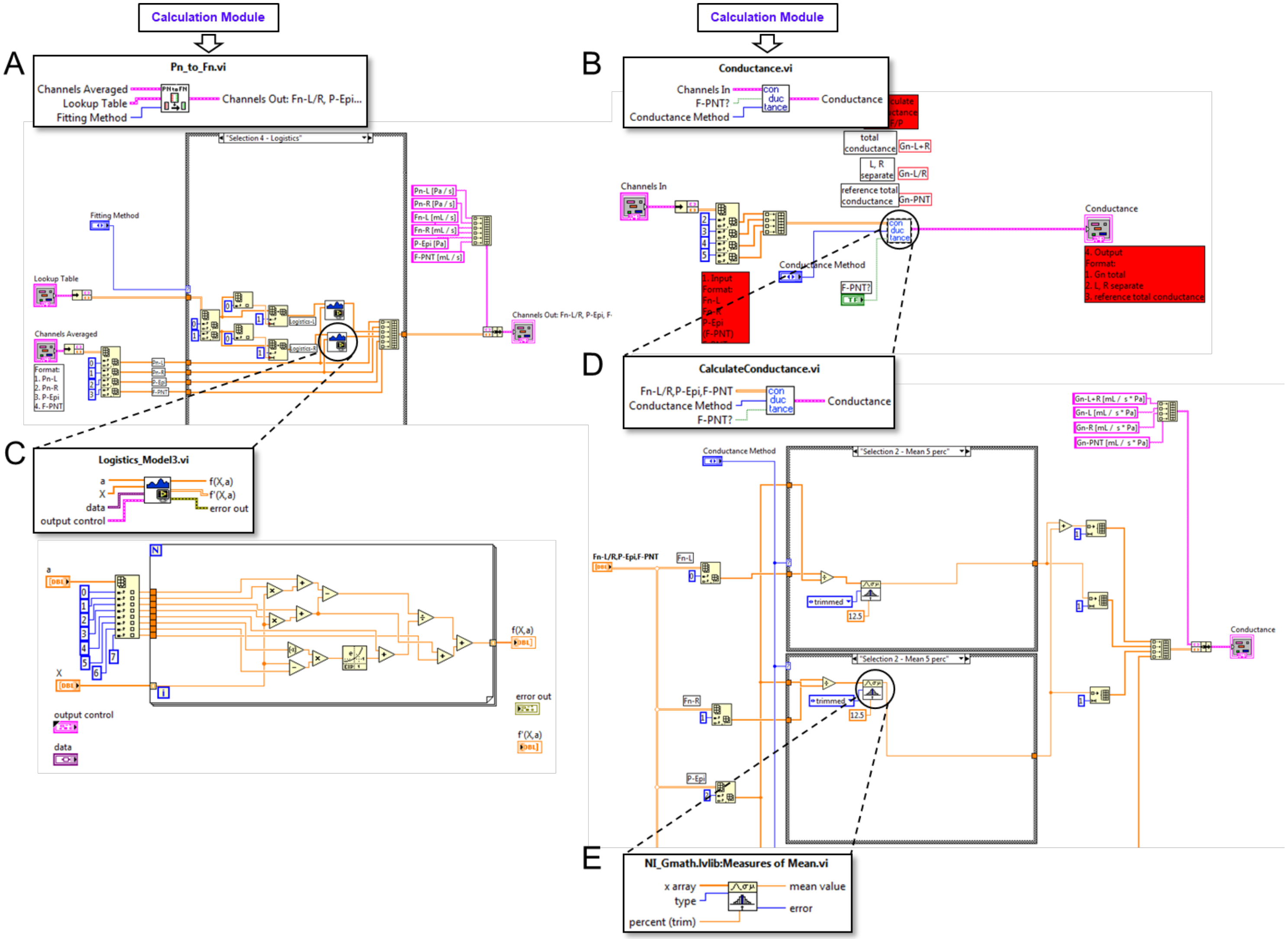

Supplementary Figure 4. LabVIEW block diagrams of two crucial signal processing steps, which are part of the second module shown in **Supplementary Figure 3**. **(A)**, 'Pn_to_Fn' VI. **(B)**, 'Conductance' VI. Both VIs contain additional nested sub-VIs, which in turn can again contain nested sub-sub-VIs as depicted in **(C)** to **(E)**.

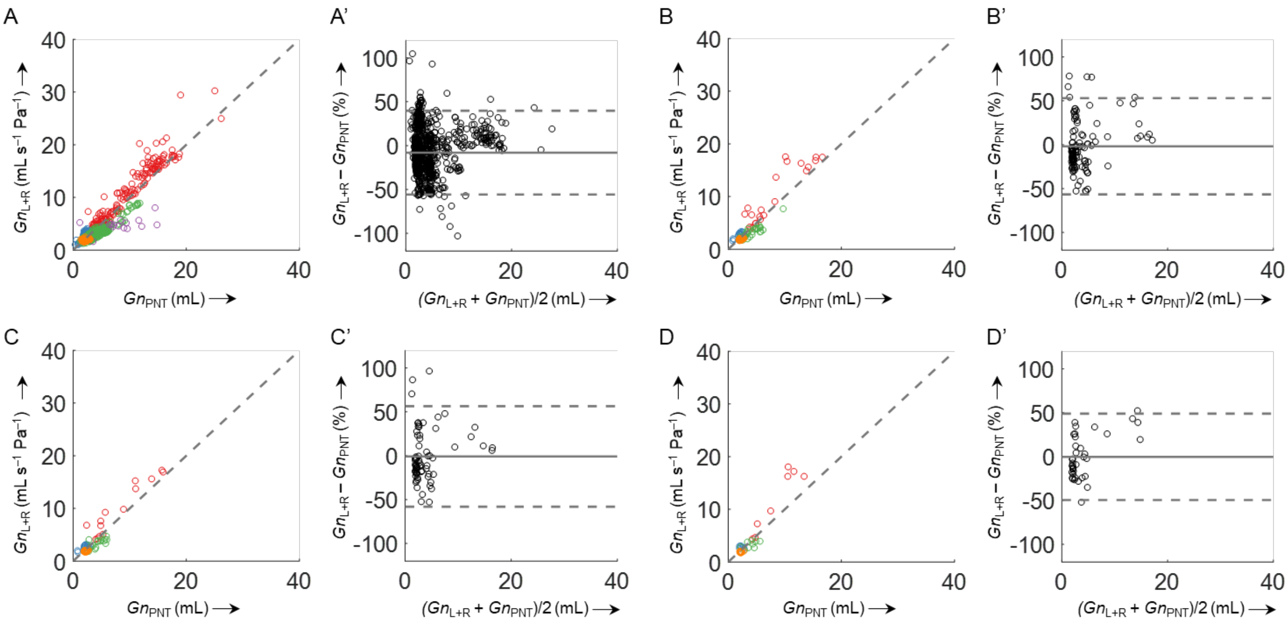

Supplementary Figure 5. Comparison of derived and measured conductance obtained by $\frac{Fn_{\mathrm{L+R}}(P_{\mathrm{Epi,max}})-Fn_{\mathrm{L+R}}(P_{\mathrm{Epi,min}})}{P_{\mathrm{Epi,max}}-P_{\mathrm{Epi,min}}}$ calculated for different time periods of averaged breath signals. Identity and corresponding Bland-Altman plots of derived total nasal conductance ($Gn_{\mathrm{L+R}}$) and Gn_{PNT} for 1 min **(A,A')**, 10 min **(B,B')**, 15 min **(C,C')**, and 30 min **(D,D')** time intervals. The different colors refer to the five analyzed datasets. The dashed gray line indicates unity. Conductance values of time intervals with VT_{Fn} or VT_{PNT} <200 mL/s or >800 mL/s and outliers ($Gn_{\mathrm{L+R}}$–Gn_{PNT} > 500%) were excluded from the comparison.

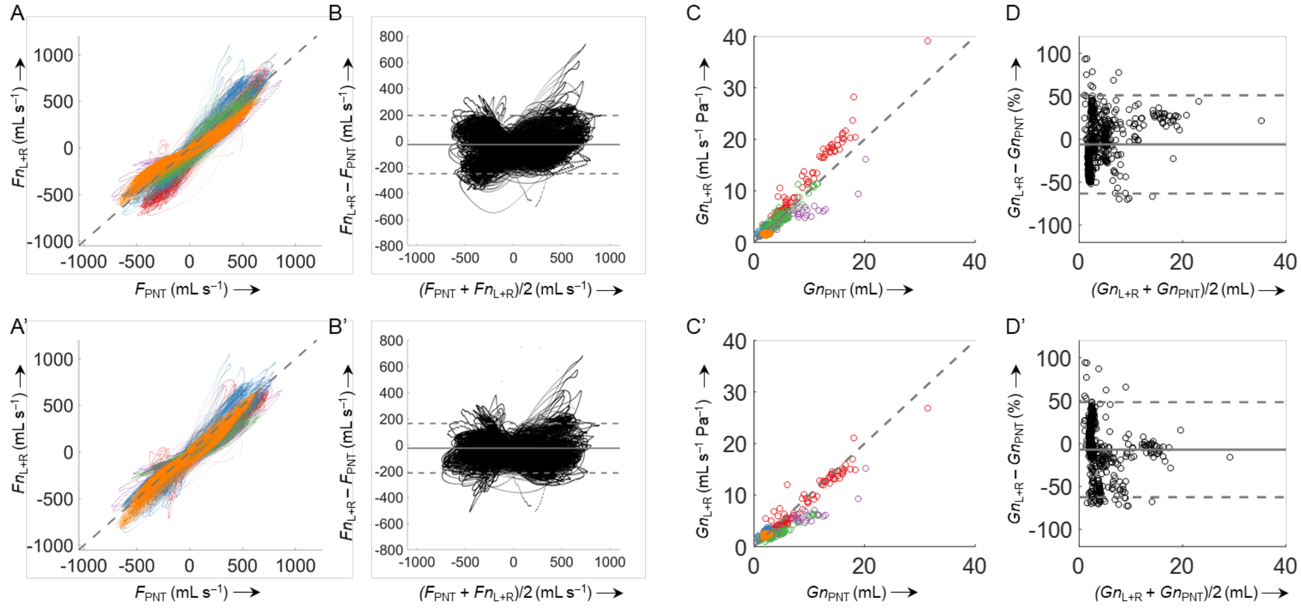

Supplementary Figure 6. Bland-Altman analysis of airflow derived solely from one calibration event, carried out either before (panels in top row) or after (bottom row) the overnight recording. **(A,A')**, Identity plots of left and right nasal pressure derived nasal airflow (Fn_{L+R}) versus values measured by a flow meter (F_{PNT}); the dashed gray line indicates identity. **(B,B')**, Bland-Altman plots of airflow; the solid line represents the mean difference (bias), dashed lines represent limits of agreement (±1.96 SD). **(C,C')**, Identity plots of derived total nasal conductance (Gn_{L+R}) and Gn_{PNT} obtained by algorithm 3; the dashed gray line indicates identity. **(D,D')** Bland-Altman plots of conductance; the solid line represents the mean difference (bias), dashed lines represent limits of agreement (±1.96 SD). Values from 5 analyzed subjects (differenct colors) were included. Averaged periods of breath with a VT <200 mL or >800 mL and outliers (Gn_{L+R}–Gn_{PNT} > 500%) were excluded from the comparison.

Supplementary Table 1. Results of Bland-Altman analysis of airflow derived solely from one calibration event as depicted in **Supplementary Figure 6(A,B,A',B')**.

Calibration event	Number of paired measurements (10^5)	Bias (mL $s^{–1}$)	Limits of agreement (mL $s^{–1}$)
Before recording	5.5	–28.7	222
After recording	5.21	–24.3	189

Supplementary Table 2. Results of Bland-Altman analysis of conductance derived solely from one calibration event as depicted in **Supplementary Figure 6(C,D,C',D')**.

Calibration event	Number of paired measurements	Bias (mL $s^{–1}$ $Pa^{–1}$)	Limits of agreement (mL $s^{–1}$ $Pa^{–1}$)	Bias (%)	Limits of agreement (%)
Before recording	550	0.1	3.6	–6.0	57
After recording	521	–0.5	2.6	–7.2	55
